# Silk Worms at Work

**Published:** 1881-08

**Authors:** 


### SILK WORMS AT WORK—INTERESTING SCENE IN A STORE WINDOW.

"Look at them nasty snaix, and ugh ! that man takes them in his hand," said a young lady to a companion, as they pressed through the crowd this morning to look in the window of the establishment of Messrs. Strawbridge & Clothier, on the corner of Market and Eighth streets, Philadelphia. This firm presents the interesting spectacle of between three and four thousand silk worms at work. A few days ago the worms were but a fraction of an inch in length; now they are from three to four and a half inches long, white in color, and just before they commence spinning the silk into a cocoon, with which they cover themselves, they become perfectly transparent. When in this condition, if one is pulled apart the silk tissues will be discovered in the centre, and will stretch for several inches. These worms have been regularly fed every few days on white mulberry leaves (*morus mullicaulus*) and grow so rapidly that the change can be seen from hour to hour. When they commence spinning they begin with a slender thread of silk and wind it around their body in a web-like texture in one continuous string; they attach themselves lightly to a leaf or some other convenient substance, and in from three to four days the cocoon is complete. They are at work in Messrs. Strawbridge & Clothier's window, and can be seen in every degree of their transition. When the cocoon is completed the worm inside is stifled and the cocoons put in hot water, which loosens and dissolves the covering with which the little casket is protected. Next, the silk threads of three or four of them are gathered together and the silk reeled off into a continuous strand of what is known as raw silk. If the moth—which would form in about ten days if the worm was allowed to remain in the chrysalis state—is allowed to come out, the cocoon is of comparatively little value; but the insect will lay about 200 eggs and then die at once. Of late years strong efforts have been made to introduce extensive silk culture into the country and to create a market for cocoons when they are produced. While, within the past year or two, the interest in this industry has enlarged considerably it has not been established firmly anywhere. Cocoons are produced in large numbers in various parts of the

United States, but most of them have to be sent abroad to be converted into raw silk, in which form the material comes back as an article of import. The efforts of those interested in the matter have been directed toward the enlargement of such a production to such an extent that manufacturers in the United States can be supplied with material for their filaments, many of which have to stand idle because of the uncertain and irregular productions.—*Philadelphia Bulletin.*

